# HCP5, as the sponge of miR-1291, facilitates AML cell proliferation and restrains apoptosis via increasing PIK3R5 expression

**DOI:** 10.1186/s40246-021-00340-5

**Published:** 2021-06-29

**Authors:** Yan Liu, Xue-Bing Jing, Zhen-Cheng Wang, Qing-Kun Han

**Affiliations:** 1 Department of Hematology, Zibo Central Hospital, No. 54 Gongqingtuan West Road, Zhangdian District, Zibo, 255000 Shandong People’s Republic of China; 2 Department of Nursing, Zibo Central Hospital, Zibo, 255000 Shandong People’s Republic of China

**Keywords:** Acute myeloid leukemia, HCP5, Apoptosis, miR-1291, PIK3R5

## Abstract

**Background:**

Acute myeloid leukemia (AML) is recognized as a hematological neoplasm with heterogenetic cytology and short-term outcome. HCP5 has been proven to be related with the pathogenesis of AML. However, the underlying mechanism of HCP5 in AML remains unclear.

**Methods:**

Clinical profiles of AML patients were downloaded from TCGA and GTEx databases. LncBase and TargetScan online tools were utilized to predict potential targets, and dual-luciferase reporter assay was performed to verify the association between miR-1291 and HCP5 or PIK3R5. Cell Counting Kit 8 and flow cytometry tests were implemented to evaluate the effects of HCP5/miR-1291/PIK3R5 axis in AML cells. Quantitative RT-PCR and Western blot were conducted to detect the expression levels of genes.

**Results:**

HCP5 and PIK3R5 were significantly increased in AML tissue samples compared with healthy controls. HCP5 facilitated AML cells viability and inhibited apoptosis. There was a positive relationship between HCP5 and PIK3R5, but miR-1291 negatively regulated PIK3R5. Overexpression of PIK3R5 enhanced the promoting effect of HCP5 in the development of AML, while weakened the suppression of miR-1291 to AML progression.

**Conclusion:**

Our findings manifested that HCP5 was remarkably upregulated in AML and upregulated HCP5 promoted the malignant behaviors of AML cells by mediating miR-1291/PIK3R5 axis, which would provide a new insight for the treatment of AML.

**Supplementary Information:**

The online version contains supplementary material available at 10.1186/s40246-021-00340-5.

## Background

As an aggressive hematological and heterogeneous malignancy, acute myeloid leukemia (AML) is the most common disease related with abnormally increased proliferation and impaired ability to differentiate of myeloid progenitor cells [1]. AML is one of the most frequent acute leukemia in adults, and its natural incidence in the world is 2.5/100,000 every year [2, 3]. Despite great progresses in AML treatment, the median overall survival of AML patients still remains only 9 months [3]. Relapse as another challenge results in that only 40% young adult and 10% aged man achieve ideal prognosis [4, 5]. Thus, it is urgently needed to identify novel biomarkers for diagnosis and prognostication in AML so as to prepare efficacious treatment solutions.

The discovery of long non-coding RNAs (lncRNAs) sheds new insights for the management of AML. There is emerging evidence that lncRNAs function as key regulators for the differentiation and maturation of myeloid, participating in regulating AML cells viability and apoptosis. For example, downregulation of HOTAIRM1 promoted Ara-C-stimulated attenuation of cell proliferation and increase of apoptosis capacity [6]. Another lncRNA UCA1 has been reported to regulate cell viability of AML cells and cell cycle [7]. Human histocompatibility leukocyte antigen (HLA) complex P5 (HCP5) was identified in 1993 and mainly expressed in immune cells with putative role in autoimmunity [8]. Several reports indicate that HCP5 exerts important roles in a variety of neoplasms. HCP5 regulates pancreatic cancer cell proliferation and apoptosis by sponging miR-214-3p and modulating HDGF [9]. Through activating AP1G1, the development of colon cancer is promoted by HCP5 [10]. Silence of HCP5 plays a tumor-repressive effect via increasing miR-128-3p in thyroid carcinoma [11]. Importantly, Lei et al. have demonstrated that HCP5 could aggravate AML by elevating cell proliferation and relieving cell cycle arrest [12]. However, the complex lncRNA/miRNA/mRNA network involved with HCP5 in AML still remains elusive.

Phosphoinositide-3-Kinase Regulatory Subunit 5 (PIK3R5) was found to be positively regulated by HCP5 based on our analysis. PIK3R5, as a member of PI3Ks, plays essential roles in cell growth, motility, and intracellular trafficking [13, 14]. Abnormal expression of PIK3R5 exerts crucial roles in ovarian cancer [15]. Meanwhile, miRNAs, as a class of short non-coding RNAs, play diverse effects in cancers via modulating genes expression at post-transcriptional levels. miR-9 is involved in the regulatory action of EVI1-induced hypermethylation in AML [16]. miR-34c-5p is used to help eradicate AML stem cells and inhibits the shedding of exosome by targeting RAB27B [17]. miR-125b participates in the non-cell-intrinsic mechanism by regulating VEGFA to promote the progression of AML [18]. After bioinformatics analysis, miR-1291 showed high relevance with HCP5 and PIK3R5 in AML, indicating that these members might construct a novel ceRNA involved in AML. Increasing publications have also suggested that miR-1291 functions as a tumor-suppressive role in renal cell carcinoma, prostate cancer, and esophageal squamous cell carcinoma [19–21]. Nevertheless, the involvement of HCP5/miR-1291/PIK3R5 axis in AML cells needs to be explored.

Herein, our study showed that PIK3R5 was highly associated with HCP5 and miR-1291 in AML cells. We investigated the implication of HCP5/miR-1291/PIK3R5 in the progression of AML. Functional in vitro analyses exhibited that HCP5 elevated AML cell viability and induced reduction of apoptosis rate as the sponge of miR-1291 through upregulating PIK3R5 expression. Taken together, our findings may open up a novel prospect for AML treatment.

## Methods

### Cell lines

Human bone marrow stromal cell line HS-5, and leukemia cell lines THP-1 and K562 used in this study were provided by the American Type Culture Collection (ATCC, Manassas, VA, USA) and incubated in Roswell Park Memorial Institute (RPMI)-1640 medium containing 10% FBS, 100 μg/mL streptomycin, and 100 U/mL penicillin with 5% CO_2_ at 37 °C.

### Transfection

Cells were first maintained in six-well plates 24 h prior to transfection until they entered logarithmic growth phase. Afterwards, cells were transfected with specific plasmids, siRNAs (si-PIK3R5: 5′-CTCAGTTCTAGCTAAATCACTAG-3′, si-HCP5: 5′-CAGCTGTAATGTGTAGTTCAATG-3′) and miR-1291 mimics (5′-UGGCCCUGACUGAAGACCAGCAGU-3′) or inhibitors (5′-ACUGCUGGUCUUCAGUCAGGGCA-3′) using Lipofectamine 2000. All bioagents were synthesized by Ribobio (Guangzhou, China). Forty-eight hours, cells were collected to be used in the further experiments.

### Detection of cell proliferation

Transfected cells were washed three times with PBS and digested using 0.25% trypsin to make single cell suspension. Then, cells (100 μL, 1000 cell/well) were inoculated in 96-well plates and cultured at 37 °C with 5% CO_2_. After maintaining for 24, 48, and 72 h, CCK-8 reagent (10 μL/well) was added, and cells were incubated for additional 1.5 h in a 5% CO_2_ incubator at 37 °C. Finally, the absorbance of cells at a wavelength of 450 nm was recorded under a micro-plate reader.

### Examination of cell apoptosis

Annexin V/PI staining was applied to measure the apoptotic capacity of cells by Annexin V-FITC/PI detection kit (Beyotime Biotechnology, Shanghai, China). Forty-eight hours after transfection, cells were collected into centrifuged tubes, centrifuged at 1000 rpm × 5 min and suspended with pre-cold PBS. After removing supernatant, transfected cells were resuspended in 1 × binding buffer. Subsequently, 100 μL cell suspension (1~5 × 10^6^/mL) was incubated in 5 mL centrifuged tubes with 5 μL Annexin V/FITC and 10 μL PI for 5 min under the dark. Cell apoptosis rate was finally evaluated by a Flowjo software.

### Luciferase activity assay

Target sequences of HCP5/PIK3R5 3′-UTR containing possible miR-1291 binding sites or not were constructed into pmirGLO vector (Promega Corporation, Madison, WI, USA), named as HCP5/PIK3R5-wild type (WT) and HCP5/PIK3R5-mutant (MUT). HCP5/PIK3R5-WT and HCP5/PIK3R5-MUT were then transfected into HEK293T cells (ATCC) with miR-1291 mimic and inhibitor. After 48-h transfection, cells were harvested to be used for the detection of luciferase activity by using Dual-Luciferase® Reporter Assay System (Promega Corporation).

### QRT-PCR

According to the guideline of manufacturers, total RNA was isolated from transfected cells by TRIzol solution and quantified using the NanoDrop 2000 spectrophotometer. Reverse transcription was conducted by the TaqMan reverse transcription kit, and qRT-PCR was implemented on ABI7500 system with SYBR Premix EX Taq kit at following reaction conditions: initially denatured at 95 °C 5min, denaturation at 95 °C 30s, annealed at 60 °C 45s, and extended at 72 °C 30s, for 39 cycles. Three parallel wells were performed for each sample and every experiment was repeated three times. The relative expression of subjects was calculated with the 2^−ΔΔCt^ method, normalized to U6 or GAPDH. The primer sequences were listed: miR-1291 F: 5′-CCTGA CTGAA GACCA GC-3′, R: 5′-GAACA TGTCT GCGTA TCTC-3′; U6: F: 5′-CGCAA ATTCG TGAAG CGTTC-3′, R: 5′-CAGGG GCCAT GCTAA TCTTC T-3′; HCP5: F: 5′-GCTGG ACGAT TCTCC TCACA CT-3′, R: 5′-CTCCT CTCCA GGCAC AGGTA AT-3′; PIK3R5 F: 5′-GTGTT GTGGT CTTTG GCTCC GA-3′, R: 5′-CCATG ACTTC GCTTC ACAGG CA-3′; GAPDH: F: 5′-CCATG GGGAA GGTGA AGGTC-3′, R: 5′-GCAGG AGGCA TTGCT GATGA-3′.

### Western blot

At first, cells were lysed in RIPA buffer with protease inhibitor on ice and the concentration of separated proteins were determined using BCA method. Then 20 μg of extracted proteins was loaded in 12% SDS-PAGE, transferred onto PVDF membranes and blocked with 5% dried skim milk in TBST for 1 h. After rinsing with TBST three times, the primary antibodies including PIK3R5 (CAT no: TA505821, OriGene Technologies, Rockville, MD, USA) and GAPDH (CAT no: 60004-1-Ig, Proteintech Group, Rosemont, IL, USA) were utilized to probe the PVDF membranes at 4 °C overnight. Next, the PVDF membranes were incubated with the secondary antibody (CAT no: SA00001-1, Proteintech Group) for 1 h at room temperature. Ultimately, membranes were developed by ECL (Beyotime Biotechnology) and the intensity of protein bands were scanned using the Quantity One software.

### Statistical analysis

Data processing was conducted with the SPSS software (version 22.0, Chicago, IL, USA) and graphs were plotted by GraphPad Prism 8.0. All results were exhibited as mean ± standard deviation (SD), and the comparisons of different groups were determined using Student’s t test or one-way ANOVA following Bonferroni post hoc test. P < 0.05 suggested that the difference was statistically significant.

## Results

### PIK3R5 is positively associated with HCP5, and their expressions are significantly increased in AML

**Fig. 1:**
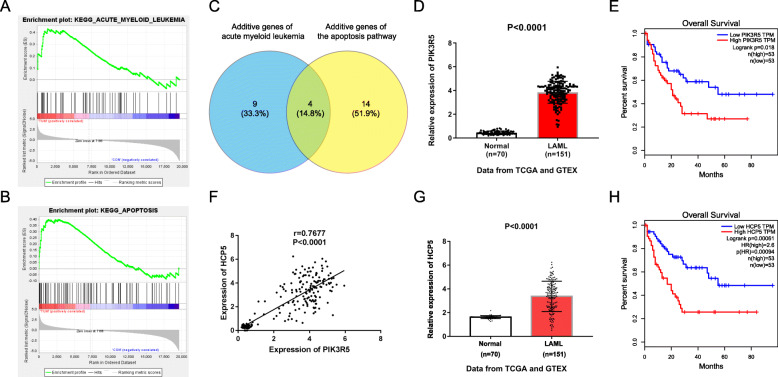
HCP5 and PIK3R5 expression were all increased in AML tissues, which correlated with unfavorable survival-term. (**A**-**B**) Gene set enrichment analysis (GSEA) was conducted with TCGA and GTEx database, and the plots revealed that a series of gene sets including acute myeloid leukemia pathway and apoptosis pathway. (**C**) Venn diagram of additive genes covered by both acute myeloid leukemia pathway and apoptosis pathway. (**D**) The expression of PIK3R5 was determined in AML tissues (n = 151) and normal controls (n = 70) on the basis of TCGA and GTEx databases. P < 0.0001. (**E**) Kaplan-Meier analysis exhibited that high PIK3R5 expression was associated with adverse overall survival. P = 0.018. (**F**) Pearson correlation analysis showed that HCP5 was positively related with PIK3R5. P < 0.0001, r = 0.7677. (**G**) HCP5 expression in AML tissue specimens (n = 151) and normal cases (n = 70). P < 0.0001. (**H**) Overall survival rates of AML patients with different HCP5 expression were detected by Kaplan-Meier method. P = 0.00094

To explore the specific mechanism of AML and identify the potential biomarker genes, we downloaded the RNA-Seq and clinical profiles of LAML from TCGA and GTEx database, including 151 tumor samples and 70 normal human bone marrow cell samples as the control group. These specimens were analyzed with GSEA, and acute myeloid leukemia and apoptosis were enriched in AML (Fig. 1A-B). Taken the intersection of genes that have an additive effect on the two pathways, and finally obtained four common genes, namely, PIK3R5, PIK3R1, PIK3CD, and NFKB1 (Fig. 1C). Next, the survival analysis showed that only PIK3R5 exhibited a significant survival value in AML; thus, PIK3R5 was selected as the candidate target. As seen from Fig. 1D, the result showed that PIK3R5 was remarkably increased in LAML samples compared with normal cases. With regard to the overall survival, AML patients with high expression of PIK3R5 had a poorer prognosis than that carrying low PIK3R5 expression (Fig. 1E). Next, co-expression analysis of PIK3R5 and lncRNA expression matrix of AML specimens indicated that PIK3R5 was co-expressed with HCP5 in AML (Fig. 1F). Furthermore, Fig. 1G showed that the expression of HCP5 was markedly higher in LAML tissues than normal controls. The survival rate of AML patients was negatively related with the expression of HCP5 (Fig. 1H). In a word, these data suggested that PIK3R5 and HCP5 may act as useful biomarkers for the treatment of AML.

### HCP5 promotes cell proliferation and inhibits apoptosis of AML cells in vitro

**Fig. 2:**
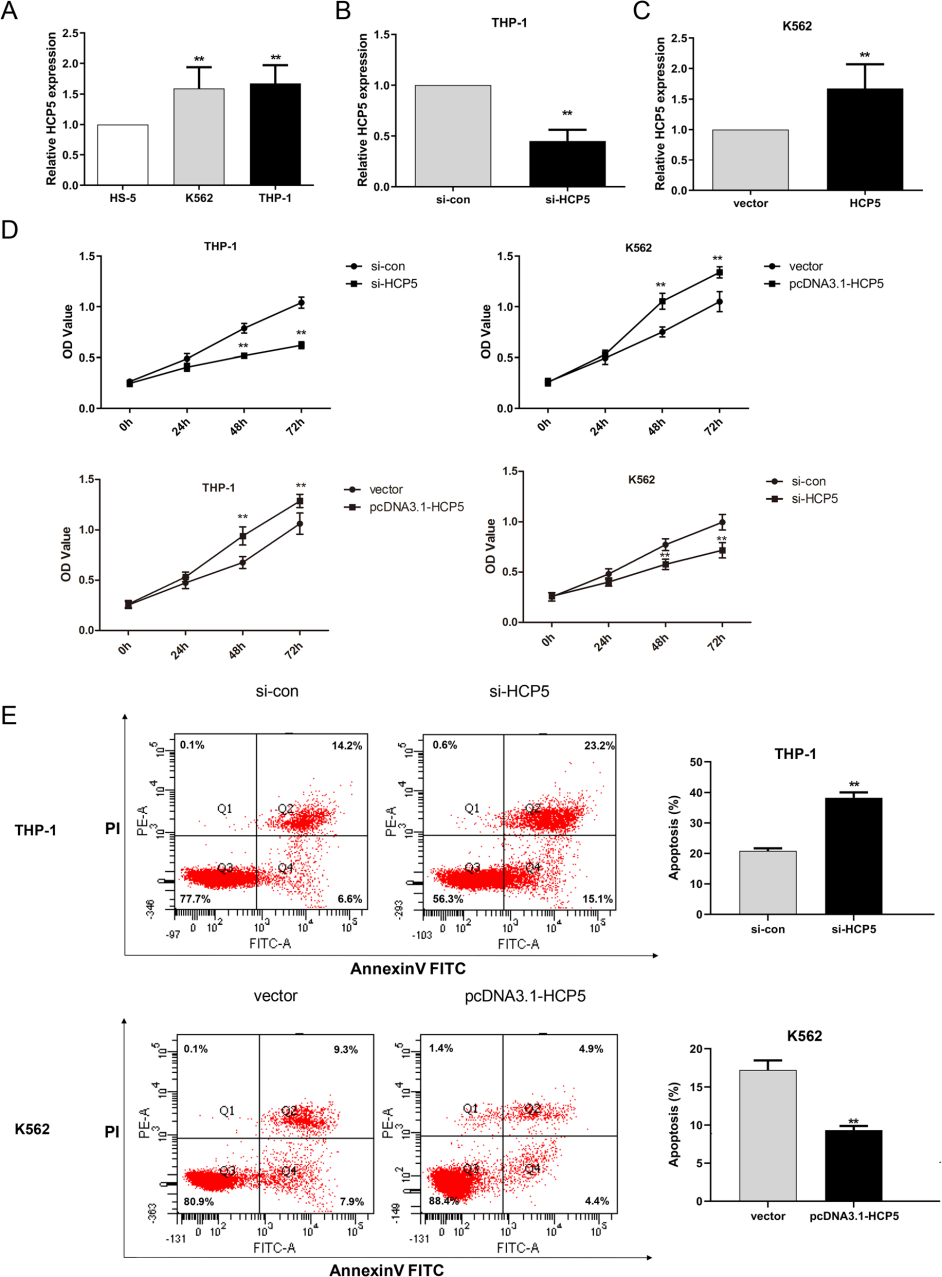
HCP5 induced the promotion of cell viability and inhibition of apoptosis in AML cells. (**A**) The expression of HCP5 in HS-5, K562, and THP-1 cells was detected by qRT-PCR. (**B**) The transfection of si-HCP5 evidently decreased the expression of HCP5 in THP-1 cells. (**C**) K562 cells transfected with pcDNA3.1-HCP5 showed an increase HCP5 level. (**D**) Treated AML cell viability was examined with CCK-8 analysis. (**E**) The rate of apoptosis in THP-1 and K562 cells was assessed using flow cytometry experiment. **P < 0.01 versus HS-5, si-con, or vector

We next conducted the qRT-PCR experiment to detect the expression levels of HCP5 in human bone marrow stromal cell line HS-5, and leukemia cell lines THP-1 and K562. Results revealed that compared with HS-5 cells, HCP5 was expressed at higher levels in both THP-1 and K562 cells (Fig. 2A). In order to investigate the exact role of HCP5 in AML cells, si-HCP5 and pcDNA3.1-HCP5 were transfected into THP-1 and K562 cells for the manipulation of HCP5 levels. As illustrated in Fig. 2B-C, HCP5 expression was significantly decreased by si-HCP5 in THP-1 cells; however, the expression of HCP5 was increased in K562 cells. The effects of HCP5 knockdown and HCP5 overexpression on cell viability were measured using CCK-8 assay, and the results manifested that downregulation of HCP5 significantly suppressed cell proliferation in AML cells, while overexpression of HCP5 remarkably elevated the proliferative ability of AML cells (Fig. 2D). To determine whether the dysregulation of HCP5 affects apoptosis of AML cells, we performed a flow cytometric test in si-HCP5-treated THP-1 cells and HCP5-overexpressing K562 cells (Fig. 2E). The data exhibited that the apoptosis rate of THP-1 cells in si-HCP5 group was higher than that in si-con group. However, in K562 cells, upregulation of HCP5 significantly attenuated the apoptosis rate compared with vector group. Collectively, these findings suggested that HCP5 promoted cell proliferation and inhibited apoptosis in AML cells.

### HCP5 sponges miR-1291, and miR-1291 directly targets PIK3R5

**Fig. 3:**
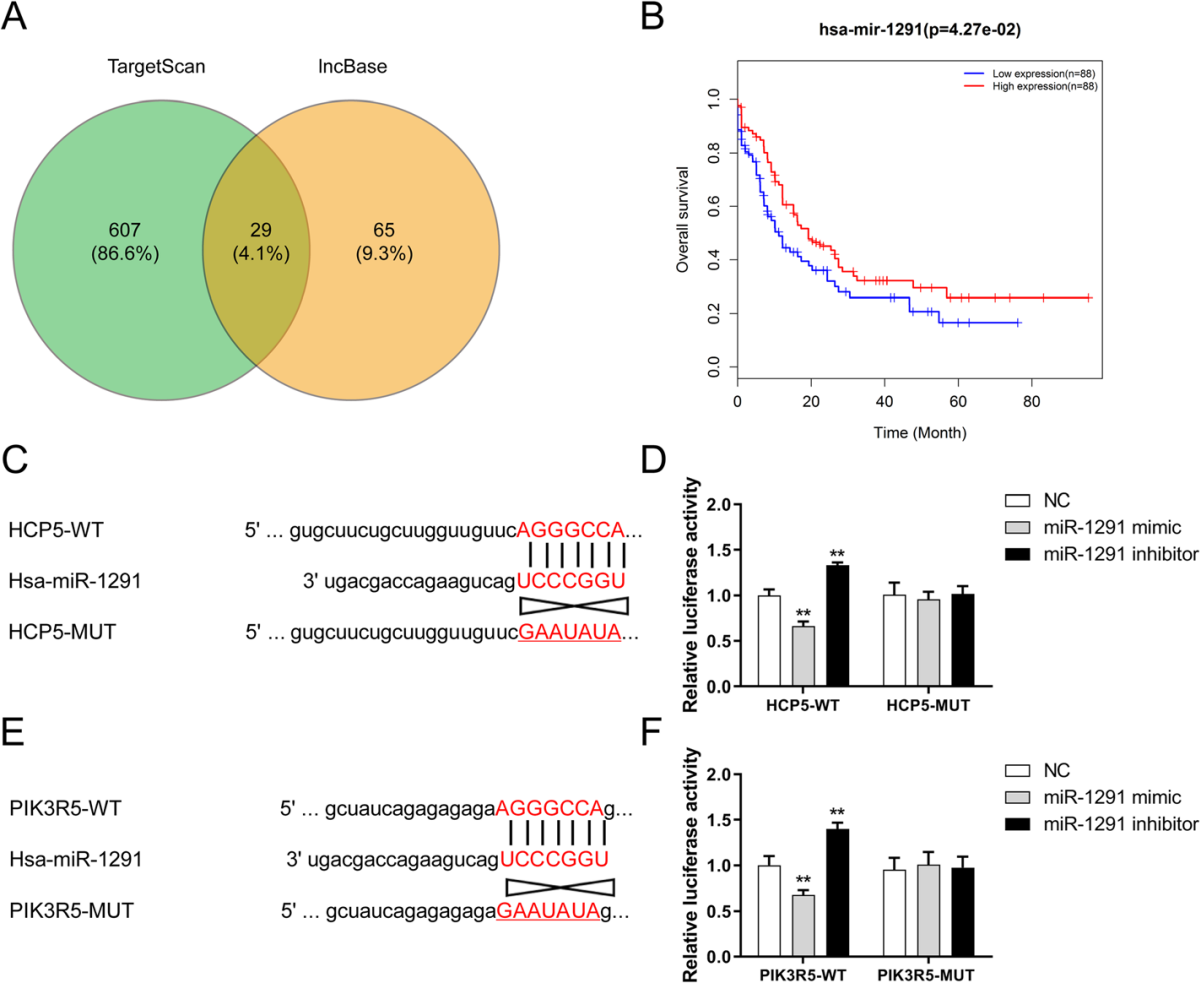
miR-1291 was sponged by HCP5 and directly targeted PIK3R5 in AML. (**A**) Venn diagram of the intersection between possible targeted miRNAs. (**B**) Overall survival analysis of AML patients with high or low miR-1291 expression. P < 0.05. (**C**-**F**) The targeting relationship between miR-1291 and HCP5 or PIK3R5. **P < 0.01 versus negative control (NC)

Bioinformatics analysis on the basis of online prediction tools lncBase and TargetScan was implemented to predict the targeted miRNAs of HCP5 and regulatory miRNAs of PIK3R5, respectively. The overlap included 29 candidate miRNAs (Fig. 3A and Additional file: Table 1). Using miRNA-seq expression data and clinical data of LAML patients derived from TCGA database for KM survival analysis, it was found that only miR-1291 had a good prognostic performance, and the survival status of the high miR-1291 expression group was significantly better than that of the low miR-1291 expression group (Fig. 3B). Sequences of shared sites between miR-1291 and HCP5/PIK3R5 were shown in Fig. 3C and E. Subsequently, a luciferase activity assay was performed to confirm their correlations among HCP5, miR-1291, and PIK3R5. The relative luciferase activity of HCP5-WT group was significantly decreased under the impact of miR-1291 mimic, and low miR-1291 expression caused by the influence of miR-1291 inhibitor markedly increased the luciferase activity of HCP5-WT group whereas the transfection of miR-1291 mimic and inhibitor did not affect the luciferase activity of HCP5-MUT group (Fig. 3D). Similarly, the luciferase activity of PIK3R5-WT was attenuated after cells transfected with miR-1291 mimic, and significantly elevated due to the low miR-1291 expression. Yet, the luciferase activity in the PIK3R5-MUT group showed no obvious difference between two treatments (Fig. 3F).

**Fig. 4:**
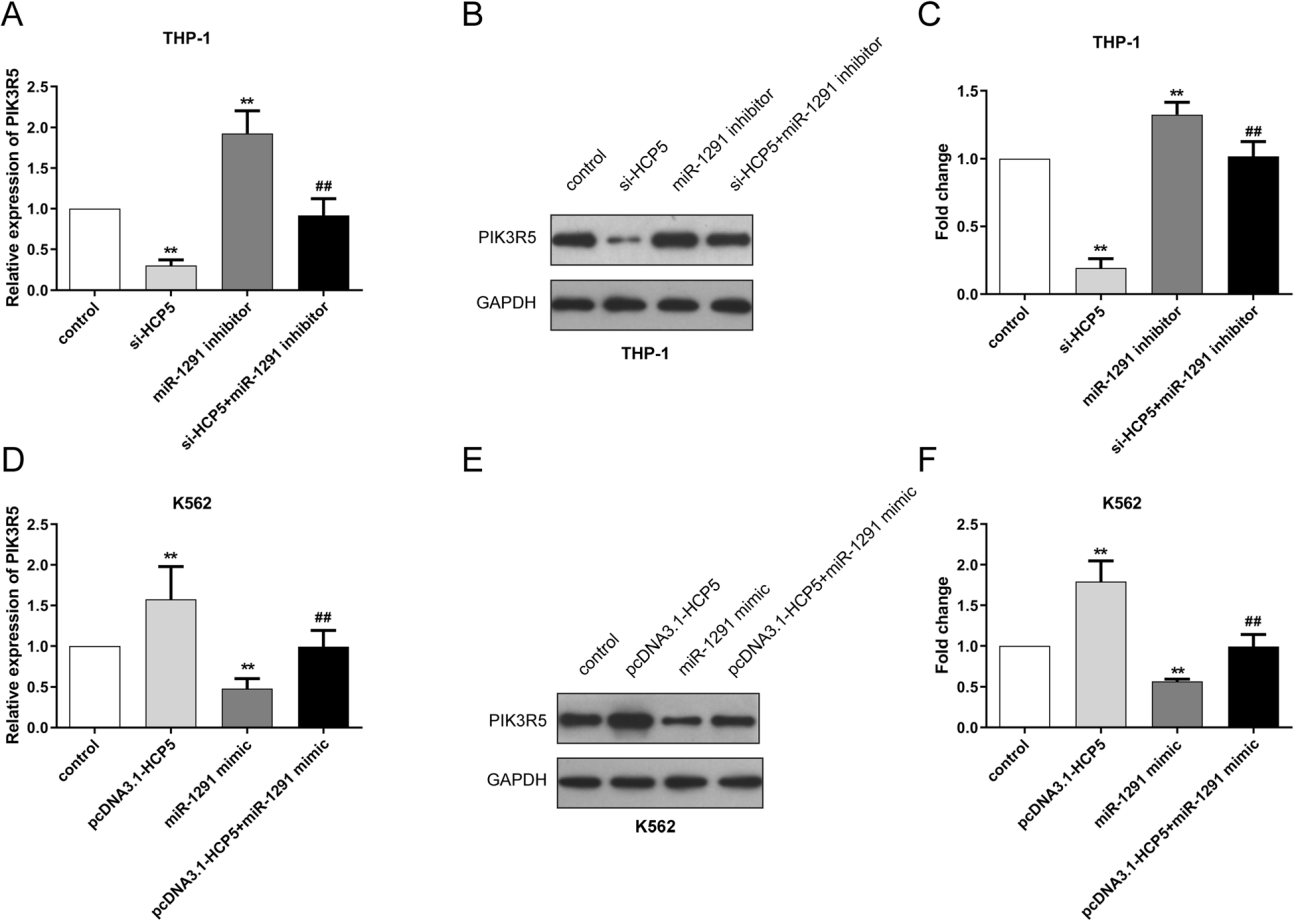
The expression of PIK3R5 was positively regulated by HCP5, whereas negatively modulated by miR-1291 in THP-1 and K562 cells. (**A**-**F**) Expression of PIK3R5 was evaluated by qRT-PCR and Western blot analyses. **P < 0.01 versus control, ^##^P < 0.01 versus miR-1291 inhibitor or miR-1291 mimic

To further verify their paired binding, qRT-PCR and Western blot experiments were implemented to evaluate the expression of PIK3R5 in AML cells after various transfections. In THP-1 cells, knockdown of HCP5 induced a decreased level of PIK3R5, while the transfection of miR-1291 inhibitor significantly promoted the expression of PIK3R5. Co-transfection of si-HCP5 and miR-1291 inhibitor canceled the impacts of individual si-HCP5 or miR-1291 inhibitor (Fig. 4A-C). On the contrary, in K562 cells, HCP5 overexpression dramatically elevated the expression of PIK3R5, but miR-1291 mimic transfection decreased the PIK3R5 expression, which were all rescued owing to the co-transfection of pcDNA3.1-HCP5 and miR-1291 mimic (Fig. 4D-F). Thus, we realized that HCP5 can directly sponge miR-1291 and PIK3R5 which may function as a target of miR-1291 in AML.

### HCP5/miR-1291 axis regulates proliferation and apoptosis of AML cells via PIK3R5

**Fig. 5:**
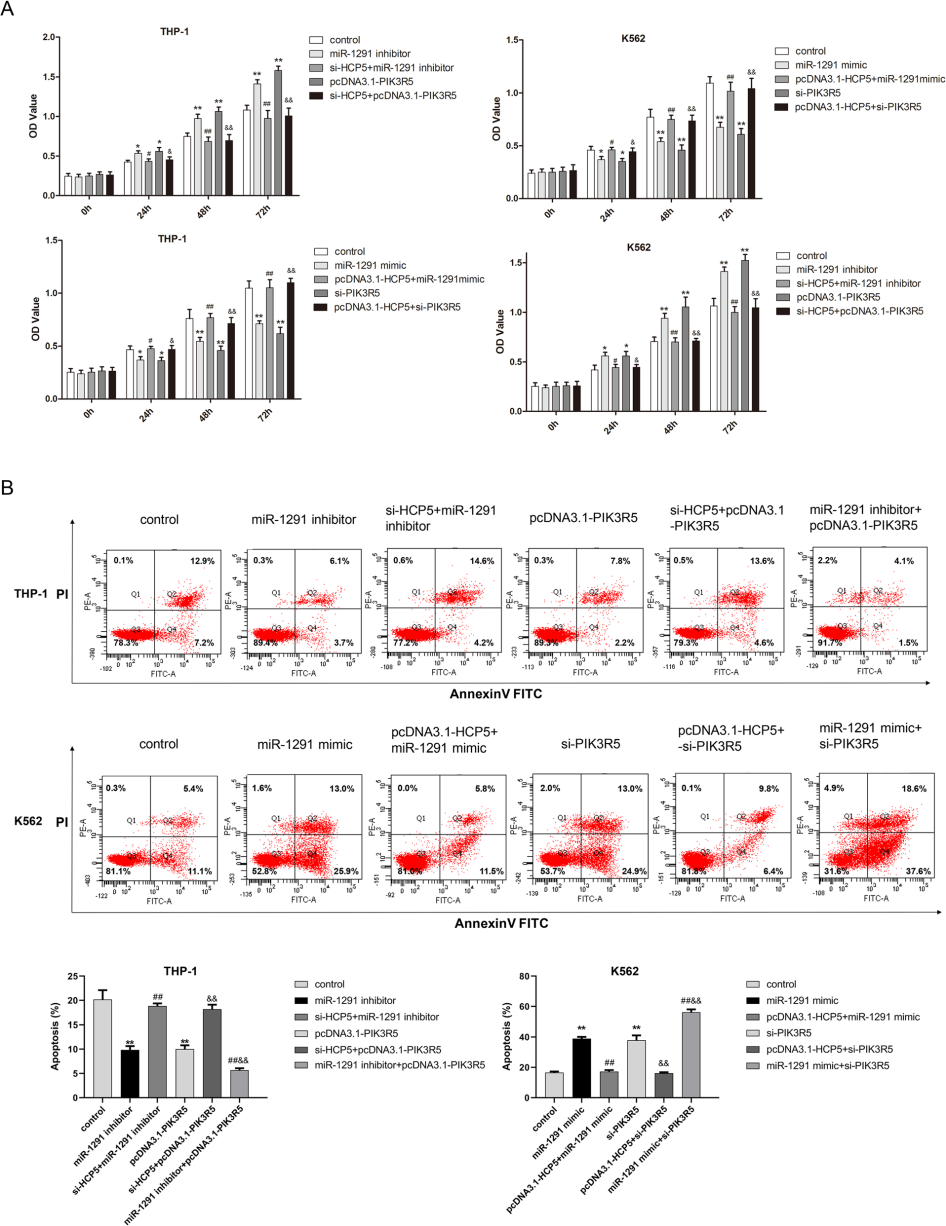
Effects of HCP5/miR-1291/PIK3R5 network on proliferation and apoptosis of AML cells. HCP5 stimulated cell viability and attenuated apoptosis rate of AML cells via regulating miR-1291/PIK3R5 axis by using (**A**) CCK-8 and (**B**) flow cytometric tests. *P < 0.05 and **P < 0.01 versus control, ^#^P < 0.05 and ^##^P < 0.01 versus miR-1291 inhibitor or miR-1291 mimic, ^&^P < 0.05 and ^&&^P < 0.01 versus pcDNA3.1-PIK3R5 or si-PIK3R5

For exploring underlying mechanism of HCP5/miR-1291 and PIK3R5 in AML cells, CCK-8 and flow cytometric assays were conducted. Results of CCK-8 analysis elucidated that miR-1291 inhibitor or PIK3R5 overexpression significantly strengthened cell viability, when compared with control group. But the transfection of si-HCP5+miR-1291 inhibitor inhibited the proliferation of AML cells relative to miR-1291 inhibitor group. The promoting effect of PIK3R5 overexpression on cell viability was latterly enhanced due to the addition of miR-1291 inhibitor. Conversely, compared with control group, miR-1291 mimic or si-PIK3R5 suppressed AML cells proliferation. Relative to miR-1291 mimic, co-transfection of pcDNA3.1-HCP5 and miR-1291 mimic partially rescued cell viability of AML cells, and the cell proliferation of si-PIK3R5 group was also promoted by the HCP5 overexpression (Fig. 5A). For apoptosis analysis, decreased apoptosis rate of THP-1 cells was induced by miR-1291 inhibitor or pcDNA3.1-PIK3R5. Silence of HCP5 overturned the suppressive effect of miR-1291 inhibitor or PIK3R5 overexpression on apoptotic capacity in THP-1 cells. Apoptosis rate of THP-1 cells treated with miR-1291 knockdown and PIK3R5 overexpression was further attenuated compared with miR-1291-silencing-THP-1 cells or PIK3R5-overexpressing-THP-1 cells. Compared with control group, overexpression of miR-1291 or PIK3R5 knockdown significantly increased apoptosis rate of K562 cells, which was reversed by the transfection of pcDNA3.1-HCP5. Compared with miR-1291 mimic or si-PIK3R5, the apoptosis rate of K562 cells transfected with miR-1291 mimic and si-PIK3R5 was further stimulated (Fig. 5B). Taken together, these above data demonstrated that HCP5/miR-1291 axis modulated the proliferation and apoptosis of AML cells through PIK3R5.

## Discussion

In the exploration, HCP5 was observed to be highly expressed in AML tissues and cells. There was a regulatory association between HCP5 and miR-1291/PIK3R5. HCP5 could induce the elevation of cell viability and suppression of cell apoptosis in AML, whereas overexpression of miR-1291 in AML cells revealed the opposite effects. We found that HCP5 functioned as a sponge of miR-1291 affecting the proliferative and apoptotic capacities of AML cells via upregulating PIK3R5.

A great deal of literatures have manifested that HCP5 acts as an important lncRNA during oncogenesis, including osteosarcoma [22], hepatocellular carcinoma [23], and breast cancer [24]. HCP5 is significantly increased in diverse tumor cells, which is highly related with prognosis, tumor formation, and metastasis [25–27]. Accumulating evidence has also showed that overexpression of HCP5 can facilitate malignant cellular behaviors of tumor cells. Chen et al. substantiated that upregulation of HCP5 stimulates cell proliferation and inhibits apoptosis in human granulosa cells [28]. Knockdown of HCP5 plays an inhibitory effect on osteosarcoma cells proliferation, invasion, and epithelial-mesenchymal transition (EMT) [22]. Notably, a previous study unraveled that the expression of HCP5 is expressed at higher levels in AML and also correlated with unideal outcomes of AML patients [12]. Our work based on the profiles of TCGA and GTEx databases showed the consistent results with the above report. In addition, CCK-8 and flow cytometric experiments disclosed that silencing of HCP5 could inhibit cell viability and promote apoptosis in AML cells, while HCP5 overexpression led to the exaltation of cell proliferation and reduction of apoptosis.

Some reports suggest that lncRNAs function as competing endogenous RNAs to modulate their target miRNAs and miRNAs which can target mRNAs, thereby affecting post-transcriptional regulation [29]. HCP5 has been reported to modulate many miRNAs expression, including miR-203 [30], miR-27a-3p [28], and miR-17-5p [31]. Herein, to further explore the relevant networks of HCP5 in AML, we screened the differential genes and performed the bioinformatics analysis. The results revealed that miR-1291 was sponged by HCP5 and directly targeted PIK3R5 in AML cells. miR-1291 displays a lower expression in various tumors and participates in carcinogenesis. Recently, miR-1291 targets the FOXA2-AGR2 signaling to inhibit the proliferation and tumorigenesis of pancreatic cancer cells [32]. miR-1291 biosynthesized in *Escherichia coli* could effectively attenuate the expression of target genes in human cancers and improve their chemosensitivity [33]. Through downregulating MED1, miR-1291 hinders cell viability and invasion in prostate cancer [34]. However, its exact role in AML has yet been illuminated. In this present investigation, high miR-1291 expression of AML patients was associated with longer survival times than that with low miR-1291 expression. Moreover, miR-1291 suppressed AML cell viability and stimulated apoptosis, which was opposite with the role of HCP5. In addition, we also found that PIK3R5 was positively regulated by HCP5, but inversely regulated by miR-1291. The results of functional in vitro experiments substantiated that the HCP5 could promote AML cell proliferation and block apoptosis via sponging miR-1291 and increasing the expression of PIK3R5.

Nevertheless, there were a few limitations needed to be deciphered. First, the accuracy of these results should be validated in other cohort with large sample size. Second, the accurate interaction among HCP5/miR-1291/PIK3R5 needs to be succumbed to more detailed investigation. Moreover, our study only focused on the in vitro analysis, which made our study relatively inadequate and the results lack of credibility.

## Conclusion

In conclusion, HCP5 and PIK3R5 were all remarkably increased in AML. The regulatory action between HCP5 and miR-1291 or PIK3R5 in AML cells was detected, which exhibited that HCP5 upregulated PIK3R5 to promote cell viability and repress apoptosis in AML cells via sponging miR-1291, thereby elevating the progression of AML. To sum up, the identification of HCP5/miR-1291/PIK3R5 network sheds some light on effective therapeutic treatment for AML.

## Supplementary Information

**Additional file 1.** Interaction miRNAs with Targetscan prediction and Lncbase prediction.

## Data Availability

All data generated or analyzed during this study are included in this published article.

## Abbreviations

AML: Acute myeloid leukemia
EMT: Epithelial-mesenchymal transition
HCP5: HLA complex P5
lncRNAs: Long non-coding RNAs
PIK3R5: Phosphoinositide-3-Kinase Regulatory Subunit 5
