# Supplementary material for: HCP5, as the sponge of miR-1291, facilitates AML cell proliferation and restrains apoptosis via increasing PIK3R5 expression
Source: Hum Genomics. 2021 Jun 29;15:38. doi: 10.1186/s40246-021-00340-5 (PMC8244151; doi:10.1186/s40246-021-00340-5)
Supplement: Supplementary file 1 — Additional file 1. Interaction miRNAs with Targetscan prediction and Lncbase prediction. [file 40246_2021_340_MOESM1_ESM.docx]

| hsa-miR-1236-3p |
| --- |
| hsa-miR-1273f |
| hsa-miR-1286 |
| hsa-miR-1291 |
| hsa-miR-211-3p |
| hsa-miR-367-5p |
| hsa-miR-4257 |
| hsa-miR-4279 |
| hsa-miR-4695-5p |
| hsa-miR-4713-3p |
| hsa-miR-4768-5p |
| hsa-miR-4779 |
| hsa-miR-5001-3p |
| hsa-miR-508-3p |
| hsa-miR-516b-5p |
| hsa-miR-608 |
| hsa-miR-629-3p |
| hsa-miR-6738-3p |
| hsa-miR-6775-3p |
| hsa-miR-6783-3p |
| hsa-miR-6797-5p |
| hsa-miR-6833-3p |
| hsa-miR-6868-3p |
| hsa-miR-6873-3p |
| hsa-miR-7114-3p |
| hsa-miR-766-5p |
| hsa-miR-769-3p |
| hsa-miR-877-3p |
| hsa-miR-942-5p |

**interaction miRNAs with Targetscan prediction and Lncbase prediction**
